# Inhibitors of riboflavin biosynthetic pathway enzymes as potential antibacterial drugs

**DOI:** 10.3389/fmolb.2023.1228763

**Published:** 2023-07-11

**Authors:** Zeyaul Islam, Pankaj Kumar

**Affiliations:** ^1^ Qatar Biomedical Research Institute (QBRI), Qatar Foundation, Hamad Bin Khalifa University, Doha, Qatar; ^2^ Department of Biochemistry, Jamia Hamdard, New Delhi, India

**Keywords:** riboflavin biosynthetic pathway, inhibitors, 3,4-dihydroxy-2-butanone 4-phosphate synthase, lumazine synthase, riboflavin synthase

## Abstract

Multiple drug resistance is the main obstacle in the treatment of bacterial diseases. Resistance against antibiotics demands the exploration of new antimicrobial drug targets. A variety of *in silico* and genetic approaches show that the enzymes of the riboflavin biosynthetic pathway are crucial for the survival of bacteria. This pathway is absent in humans thus enzymes of the riboflavin biosynthetic pathway are emerging drug targets for resistant pathogenic bacterial strains. Exploring the structural details, their mechanism of action, intermediate elucidation, and interaction analysis would help in designing suitable inhibitors of these enzymes. The riboflavin biosynthetic pathway consists of seven distinct enzymes, namely, 3,4-dihydroxy-2-butanone 4-phosphate synthase, GTP cyclohydrolase II, pyrimidine deaminase/reductase, phosphatase, lumazine synthase, and riboflavin synthase. The present review summarizes the research work that has been carried out on these enzymes in terms of their structures, active site architectures, and molecular mechanism of catalysis. This review also walks through small molecule inhibitors that have been developed against several of these enzymes.

## Introduction

Riboflavin (also known as vitamin B2) is the originator for flavocoenzymes, flavin mononucleotide (FMN), and flavin adenine dinucleotide (FAD), two necessary cofactors that play key roles in various crucial cellular functions (Muller, 2018). They are involved in a wide variety of redox reactions (single as well as two electron transfer reactions) that are vital to energy metabolism essential for all living organisms (Mansoorabadi et al., 2007; Muller, 2018). Apart from their involvement in redox reactions, they also take part in a remarkable number of non-redox processes including DNA damage repair, circadian clock regulation, signal transduction, nitrogen fixation, light sensing, and bioluminescence (Salomon et al., 2001; Bornemann, 2002; Thompson and Sancar, 2002; Wijnen and Young, 2006; Macheroux et al., 2011). Lumazine binding proteins, which can bind riboflavin, FMN, 6, 7-dimethyl-8-ribityllumazine non-covalently as a fluorophore, take part in the bioluminescence process as an optical transponders in several bacterial species (Lee, 1993; Petushkov and Lee, 1997). This astonishing photochemical reaction reveals the remarkable flexibility of the isoalloxazine chromophore, in that the flavin cofactors side chains assist mainly as anchors that locked the binding of the fluorophore to the cognate proteins. Flavin cofactors also participate to form complex catalytic centers, sometimes including more than one coenzyme, various flavin adducts and/or other cofactors such as iron-sulfur clusters (Roberts et al., 2003; Hudson et al., 2005; Cheng et al., 2006).

**FIGURE 1:**
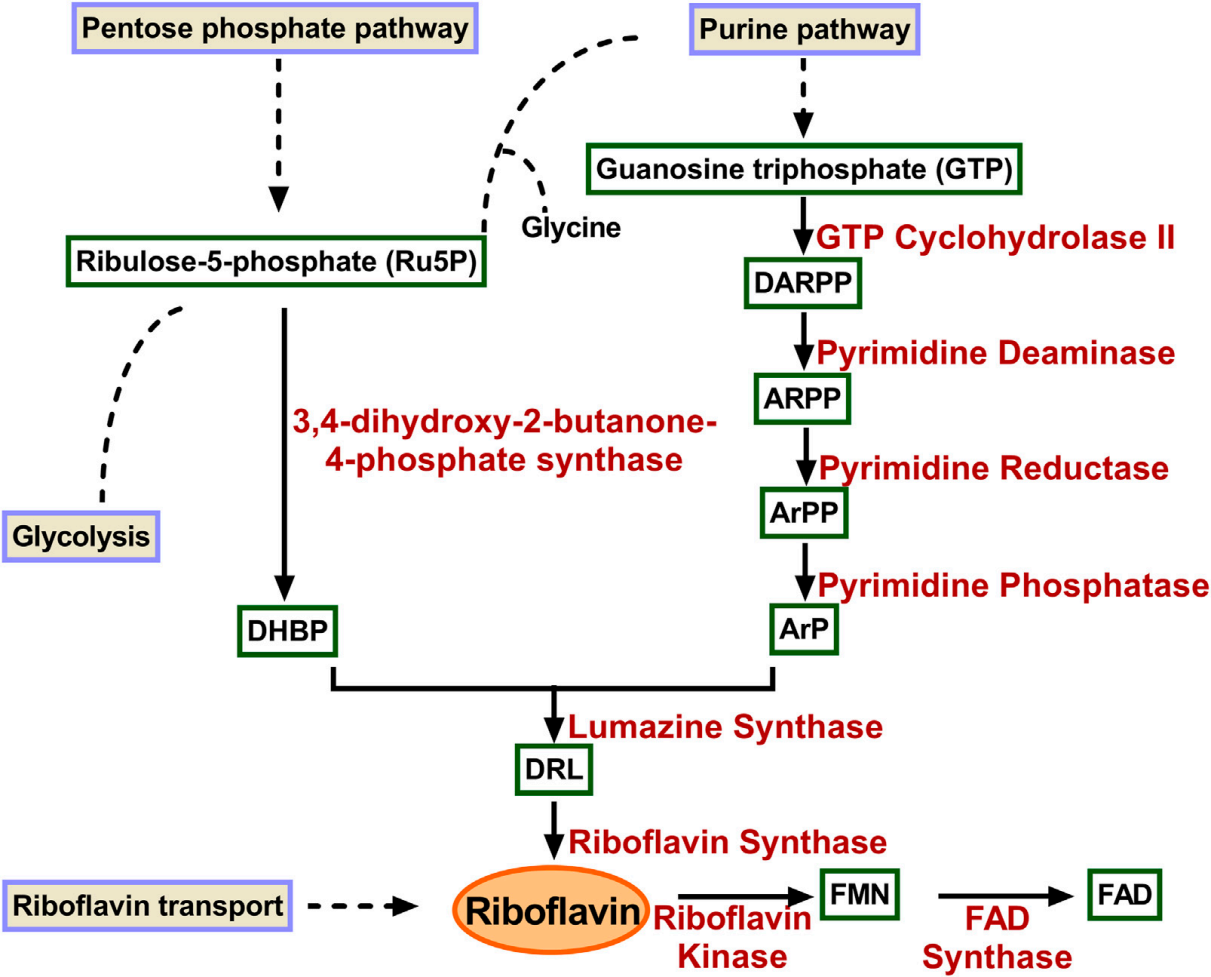
Enzymes involved in riboflavin biosynthetic pathway. GTP is converted to ArP through four reactions catalysed consecutively by GTP cyclohydrolase II, pyrimidine deaminase, pyrimidine reductas, and pyrimidine phosphatase. Similarly, DHBP is formed Ru5P catalyzed by 3,4-dihydroxy-2-butanone-4-phosphate synthase. The two products condensed to form DRL in a reaction catalyzed by lumazine synthase. Riboflavin synthase converts two molecules of DRL to riboflavin and ArP. DARPP, 2,5-diamino-6-ribosyl-amino-4(3H)pyrimidinedione 5′-phosphate; ARPP, 5-amino-6-ribosyl-amino-2.4(1H, 3H)pyrimidinedione 5′-phosphate; ArPP 5-amino-6-ribitylamino-2.4-(1H, 3H)-pyrimidinedione-5′-phosphate; ArP 5-amino-6-ribitylamino-2.4-(1H, 3H)-pyrimidinedione; DHBP 3,4-dihydroxy-2-butanone-4-phosphate; DRL 6,7-dimethyl-8-ribityl-lumazine; FMN, flavin mononucleotide; FAD, flavin adenine dinucleotide.

Riboflavin is biosynthesized *de novo* in microorganisms and plants, whereas in animals they depend on dietary supplements. The riboflavin biosynthetic pathway was extensively studied and consists of seven distinct enzymes catalyzing the reaction, starting with one guanosine triphosphate (GTP) and two molecules of ribulose 5-phosphate (Ru5P) as the initial precursors (Figure 1). GTP is transformed into 2,5-diamino-6-ribosylamino-4(3H)- pyrimidinedione 5′-phosphate (DARPP) by GTP cyclohydrolase II. The bifunctional enzyme deaminase/reductase catalyses the deamination and subsequent reduction to convert DARPP to 5-amino-6-ribitylamino-2.4(1H, 3H)-pyrimidinedione 5′-phosphate (ArPP). ARPP is dephosphorylated by yet unknown phosphatase, resulting in 5-amino-6-ribitylamino-2.4(1H, 3H)-pyrimidinedione (ArP). Along the parallel line, 3,4-dihydroxy-2-butanone-4-phosphate synthase (DHBPS) catalyzes the conversion of ribulose-5-phosphate (Ru5P) to 3,4-dihydroxy-2-butanone 4-phosphate (DHBP). The resulting products ArPP and DHBP are condensed by lumazine synthase (LS) to form 6,7-dimethyl-8-ribityllumazine (DRL) with the release of inorganic phosphate. The final step involves an unusual dismutation reaction of two DRL, converting them into one riboflavin and one molecule of ArP. This reaction encompasses the transfer of a four-carbon unit between two identical substrates and the reaction is catalyzed by riboflavin synthase (RS). ArP formed in the final reaction is recycled back and used as a substrate by LS (Foor and Brown, 1975; Burrows and Brown, 1978; Volk and Bacher, 1991; Kis et al., 1995; Fischer and Bacher, 2008; 2011; Ladenstein et al., 2013).

Identification and evaluation of novel drug targets drive the advancement of new inhibitors against pathogenic microbial strains resistant to common antibiotics. Several *in silico* approaches were engaged to investigate genomic data to identify potential drug targets that are critical for the survival and virulence of bacterial pathogens and with no counterpart in humans or other animal proteins (Gupta et al., 2019). Several approaches including a whole-genome transposon random approach to ascertain the critical gene set found in various branches of life and were applied to the genomes of *Haemophilus influenza*, *Mycobacterium tuberculosis*, *Pseudomonas aeruginosa*, *E. coli* and *Mycoplasma genitalium* (Akerley et al., 2002; Gerdes et al., 2002; Lamichhane et al., 2003; Sassetti et al., 2003; Glass et al., 2006; Liberati et al., 2006). Gene deletion techniques were also used for defining important genes in *Bacillus subtilis* and *Escherichia coli* (Kobayashi et al., 2003; Baba et al., 2006). These studies suggested that the enzymes of the riboflavin biosynthesis pathway are vital and can be used as drug targets.

Plants and many microorganisms including bacterial pathogens can manufacture riboflavin *de novo*, but animals do not have this ability, which they fulfill through their diet. Bacterial pathogens especially the Gram-negative bacteria like *M. tuberculosis* and *Salmonella typhimurium* stringently need endogenous riboflavin due to the absence of a riboflavin uptake system from the environment (Wang, 1992; Dahl et al., 2004; Long et al., 2010). Hence, the enzymes of the riboflavin biosynthetic pathway could be measured as selective therapeutic targets. Various research works reinforced the significance of *de novo* riboflavin biosynthesis in several pathogenic bacterial antibiotics-resistant microbes and enzymes of this pathway as therapeutic targets. In fact, it has also been established that riboflavin biosynthetic genes and enzymes are virulence factors in *Salmonella enterica* (Becker et al., 2006; Rollenhagen and Bumann, 2006). Hence, the development of potential drugs against the enzymes involved in the riboflavin (vitamin B2) biosynthetic pathway opens a fresh line of attack for the treatment of bacterial infections. Designing novel antimicrobial drugs that selectively target pathogens are immediately required to tackle the multidrug resistance that arises in various pathogens.

### 3,4-Dihydroxy-2-butanone 4-phosphate synthase (DHBPS)

DHBPS is one of the first enzymes involved in the riboflavin biosynthetic pathway and converts Ru5P to DHBP and formate (Figure 1). DHBPS can exist as a single enzyme or it can form a bi-functional enzyme. It combined with GTP cyclohydrolase II (GCH II) to form a bi-functional enzyme, although DHBPS and GCH II can exist as two independent enzymes. The catalytic mechanism proposed for DHBPS involves several intermediate steps including skeletal rearrangement and fragmentation. These intermediate stages culminate with the release of formate and stable endiol intermediate that undergoes protonation with the formation of DHBP (Volk and Bacher, 1990; Richter et al., 1992). The chemical transformation includes a skeletal rearrangement and proposes a central role for acid/base catalysis with substrate/intermediates interaction requires divalent metal ion Mg^2+^ (Volk and Bacher, 1990). DHBPS enzyme class from various microorganisms has a primary sequence length of 204–233 amino acid residues and with 25%–60% sequence homology.

**FIGURE 2:**
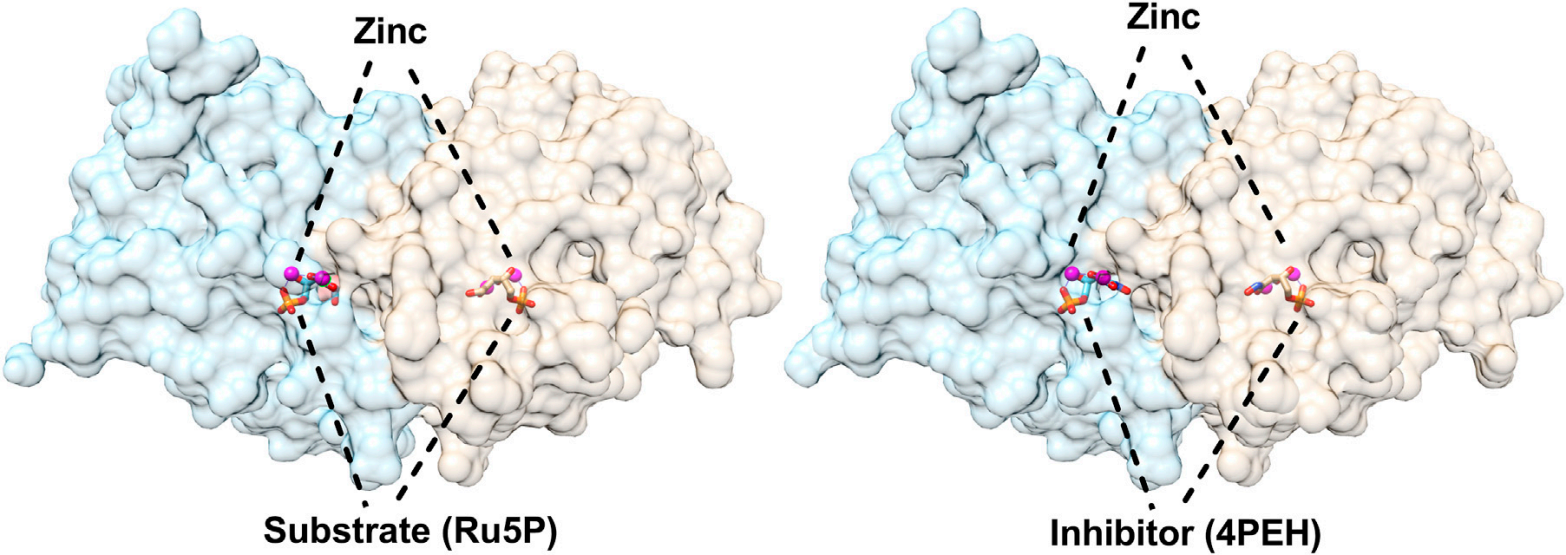
Structures of 3,4-dihydroxy-2-butanone 4-phosphate synthase (DHBPS) from *V. cholera*. Crystal structure of DHBPS in complexed with substrate (Ru5P) and metal ions (PDBID: 4P8E) showing the active site architecture (left). Similarly, crystal structure of DHBPS in complexed with inhibitor (4PEH) and zinc ions (PDBID: 4P6P) highlighting the binding of inhibitor and metal (right).

The three-dimensional structures of DHBPS were solved from *E. coli*, *M. grisea*, *M. jannaschii*, *Candida albicans*, *S. typhimurium*, *M. tuberculosis*, and *V. cholera* (Liao et al., 2001a; Liao et al., 2002; Steinbacher et al., 2003; Echt et al., 2004; Kumar et al., 2010; Singh et al., 2011; Islam et al., 2015). All of the structures solved to date indicate that DHBPS exists as a homodimer and present a characteristic alpha + beta fold containing mainly beta strands with a complex linkage. The homodimeric nature of DHBPS creates two active surfaces of the enzyme and is located at the interface between two subunits in the vicinity of the residues conserved among species. The surface of each active site is primarily shaped by one monomeric unit with an additional surface being created by neighboring monomeric unit residues (Figure 2). The active pocket of DHBPS was identified by crystallographic studies from the archaeon *M. jannaschii* and *C. albicans* in a complex with ribulose-5-phosphate and metal ions (Steinbacher et al., 2003; Echt et al., 2004) and are highlighted in Figure 2. Structures available till date suggest a degree of flexibility in the active cavity, especially at the loop regions. The DHBPS structure shows two different conformations of loop present near the active site. It can be either open or closed depending on the substrate availability. An open conformation exists in the absence of substrate where these loops points away from the active site cavity, while it closes up in the presence of substrate or substrate along with metals, which are required for the completion of catalysis, as a number of residues from these loops interact with substrate and metals (Liao et al., 2002; Steinbacher et al., 2003; Kumar et al., 2010; Islam et al., 2015). The substrate (Ru5P) present in the active site cavity also shows flexibility in its conformation. Moreover, the divalent metal ions (“dimetal center”) present at the active site (Figure 2) are crucial for the catalytic activity of DHBPS and are proposed to be involved in substrate and intermediates stabilization (Liao et al., 2002; Steinbacher et al., 2003; Islam et al., 2015). The detailed investigation of DHBPS structures in complexes with several metals highlighted the breathing of metal or shift in the position of two metals during the course of the reaction (Liao et al., 2002; Islam et al., 2015). Recently, two transient intermediates were identified using time-dependent structural studies and helps in defining the mechanism of DHBPS (Kenjić et al., 2022).

### GTP cyclohydrolase II (GCH II)

GCH II converts GTP into DARPP (2,5-diamino-6-ribosylamino-4(3H)-pyrimidinedione 5′-phosphate), formate, and inorganic pyrophosphate with the aid of divalent metal ion (Foor and Brown, 1975; Foor and Brown, 1980; Ritz et al., 2001). Formate is released from the imidazole ring while pyrophosphate is released from the nucleotide precursor affording the formation of DARPP (Foor and Brown, 1980; Ren et al., 2005). Biochemical studies suggested the role of zinc ion, which plays a crucial part in guanine ring opening although it is not needed for inorganic pyrophosphate release (Kaiser et al., 2002). The GCH II has no significant amino acid sequence similarity with GCH I and differs in functional state. The two GTP cyclohydrolase have completely different oligomeric states, while GCH II exists as a homodimer, GCH I forms as a homodecamer (Nar et al., 1995).

**FIGURE 3:**
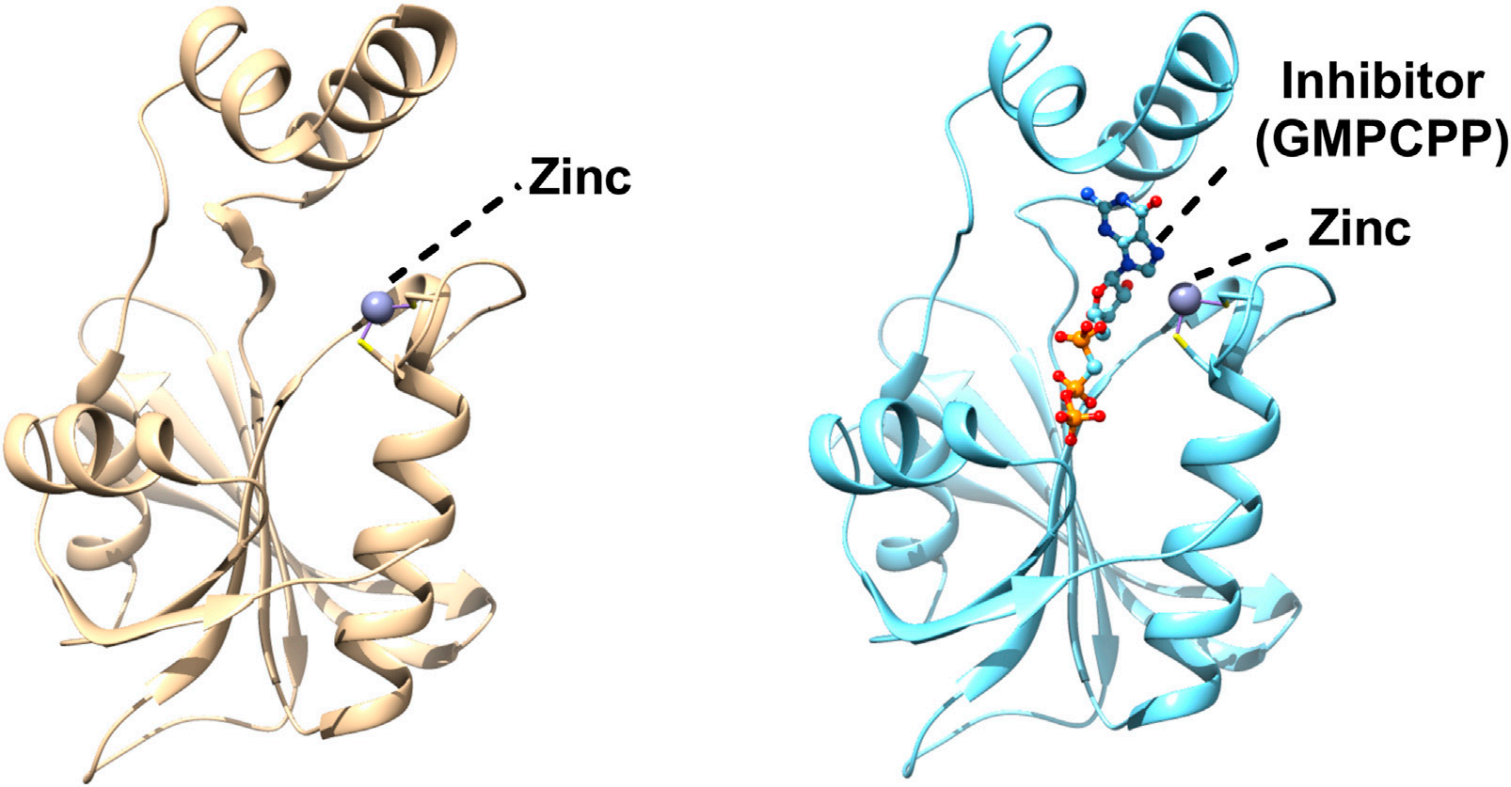
Structures of *Escherichia coli* GTP cyclohydrolase II (GCH II). Cartoon representation of *Escherichia coli* GCH II in apo form (PDBID: 2BZ1) revealing the intrinsic metal binding (left). Crystal Structure of *Escherichia coli* GCH II in complex with GMPCPP and Zinc (PDBID: 2BZ0) enlightening the inhibitor binding (right). Zinc is shown as grey ball.

Structures of GCHII in apo as well as in complex form have been solved from *E. coli*, and *Helicobacter pylori* (Ren et al., 2005; Yadav and Karthikeyan, 2015). It has an alpha/beta fold which mainly consists of a central core of antiparallel β-sheets connected by loops and helices (Figure 3). The three dimensional structure of *E. coli* GCH II was also solved with GMPCPP (substrate analogue), which showed the main residues that form the active site cavity and participate in substrate interaction. No major conformational changes were observed between the apo and holo forms of the enzymes with only a small deviation of the side chains of the residues involved in the binding of the substrate or substrate analogue (Ren et al., 2005). A zinc ion was bound intrinsically to the GCHII and is coordinated through three cysteines (Figure 3). Loss of any one of the three cysteines is enough to bring to an end the binding as well as its enzymatic function (Kaiser et al., 2002). The active region of GCH II contains substrate, GMP and zinc ion where the metal coordinated by three cysteines of a CX2GX7CXC motif. The metal was spaning the α and β phosphate moieties of the triphosphate motif and interacted with the amino acid residue of the protein (Figure 3). The complex structure also highlighted Arg128 interaction with the α-phosphonate for pyrophosphate release and formation of the proposed covalent guanylyl-GTP cyclohydrolase II intermediate (Ren et al., 2005). The structure of GCHII from *H. pylori* is similar to *E. coli*, although it is without any intrinsically bound zinc ion, probably exhibiting the inactive state of GCHII (Ren et al., 2005; Yadav and Karthikeyan, 2015).

### Pyrimidine deaminase/reductase

DARPP formed by GCHII is transformed into ArPP (5-amino-6-ribitylamino-2.4(1H, 3H)-pyrimidinedione 5′-phosphate) in two steps comprising the deamination of the pyrimidine ring and reduction of the ribosyl side chain. These two reactions are catalyzed by bifunctional pyrimidine deaminase/reductase enzyme (Richter et al., 1997). The order of the two processes is different in various organisms studied. In bacteria and plants, deamination precedes reduction; however, reduction precedes deamination in yeast and archaea (Burrows and Brown, 1978; Nielsen and Bacher, 1981; Fischer et al., 2004). The bifunctional enzyme has a deaminase domain at the N-terminal, which contains a zinc ion, and an NADPH-binding reductase domain at the C-terminal. The reductive reaction requires the NADPH in reduced form and deaminase activity abolition does not disturb the reductase activity (Magalhães et al., 2008).

**FIGURE 4:**
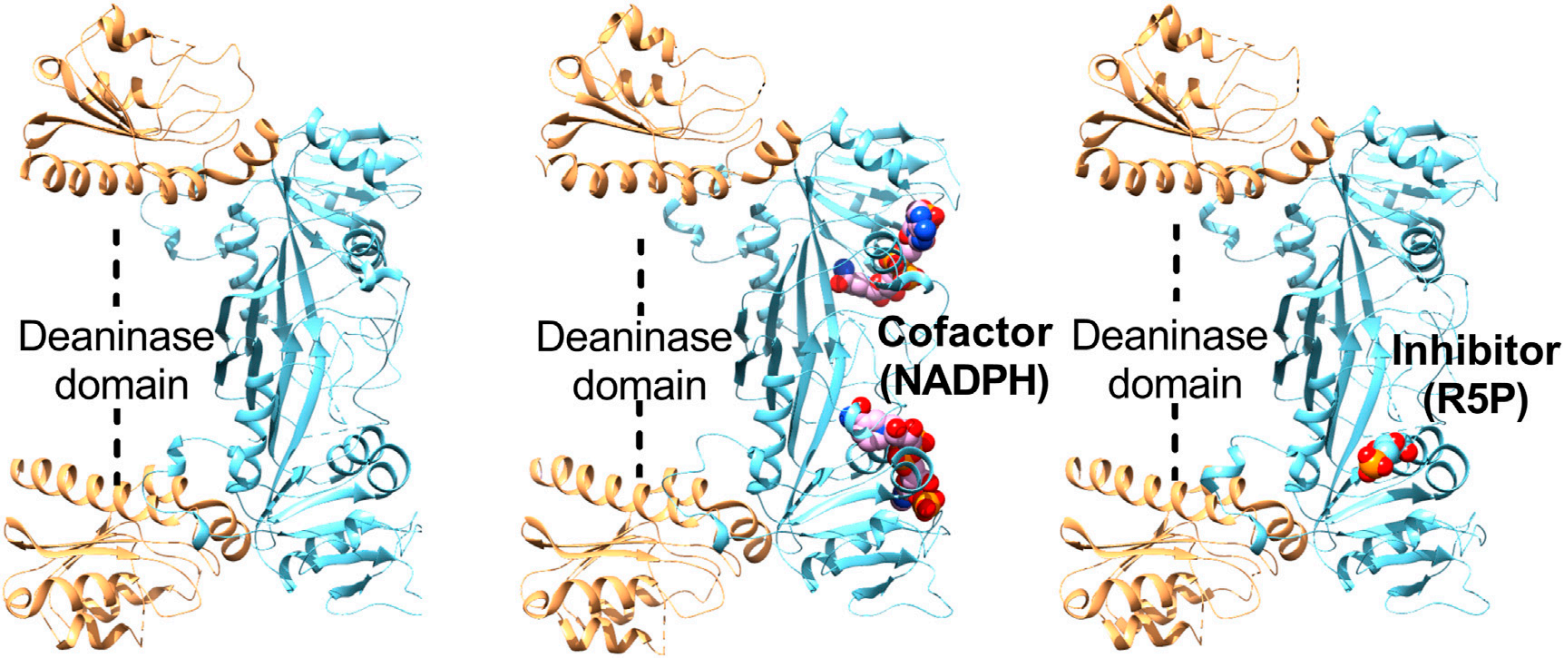
Structures of *Escherichia coli* bifunctional deaminase/reductase. Cartoon representation of the crystal structure of *Escherichia coli* deaminase/reductase in apo form (PDBID: 2G6V) underlining the domain organization and their relative orientation (left). The crystal structure of *Escherichia coli* bifunctional deaminase/reductase in complex with cofactor, NADPH (PDBID: 2O7P) binding in the active site of the reductase domain (middle). Structure of *E. coli* deaminase/reductase in complex with a substrate analogue, ribose 5-phosphate (R5P), bound to the active site of the reductase domain (right).

The x-ray crystal structure of bifunctional deaminase/reductase from *E. coli*, *B. subtilis*, *M. jannaschii* and *Acinetobacter baumannii* has been solved with several complexes (Chatwell et al., 2006; Chen et al., 2006; 2009; Stenmark et al., 2007; Dawson et al., 2013). It forms as a homodimer in *E. coli*, while exists in a homotetrameric form in *B. subtilis* (Chen et al., 2006; Stenmark et al., 2007). Each monomer is made up of two separate functional domains, comprises of an N-terminal deaminase domain and a C-terminal reductase domain with a short linker region dividing these domains (Figure 4). The N-terminal deaminase domain contains beta-sheet surrounded by alpha-helices with central portion dominated by mixed beta-strands. Similarly, the reductase domain mainly consists of beta-sheet with seven parallel strands and a β-hairpin. The deaminase domain occupies the far end and the homodimeric interface is primarily constructed by two consecutive segments of the reductase domain. The deaminase domain binds zinc with tetrahedral coordination by a zinc-binding motif having two cysteines, one histidine, and a water molecule (Dawson et al., 2013). The binding of NADP occurred at the surface of the reductase domain in a very extended conformation (Figure 4) with slight variations at the nicotinamide ring (Stenmark et al., 2007). The reductase active pocket was covered by the loop, which interacts with the cofactor and substrate analogue in the binary complexes (Ren et al., 2005). The substrate/substrate analogue ribose 5-phosphate binds to subunit A of the enzyme but does not bind to subunit B (Figure 4). A ternary complex of the reductase domain was created by a combination of the substrate with the ribitylimino intermediate and cofactor NADP binary structures (Chen et al., 2009).

### Lumazine synthase (LS)

LS facilitates the condensation of two substrates, namely, DHBP and ArPP, leading to the biosynthesis of DRL. The subsequent dismutation reaction of two DRL converts it into one riboflavin and one ArPP, catalyzed by riboflavin synthase (RS). Numerous structural, biophysical and mechanistic studies were conducted to understand the mechanism of the reaction carried out by LS. The enzyme-catalyzed reaction was anticipated to have several intermediary steps and start with the substrate binding, nucleophilic attack, formation of Schiff base intermediate, proton abstraction, and phosphate elimination with subsequent ring closure. Finally, the release of water would terminate the reaction, resulting in product creation in the form of DRL (Kis et al., 1995; Bacher et al., 1996; Zhang et al., 2003).

**FIGURE 5:**
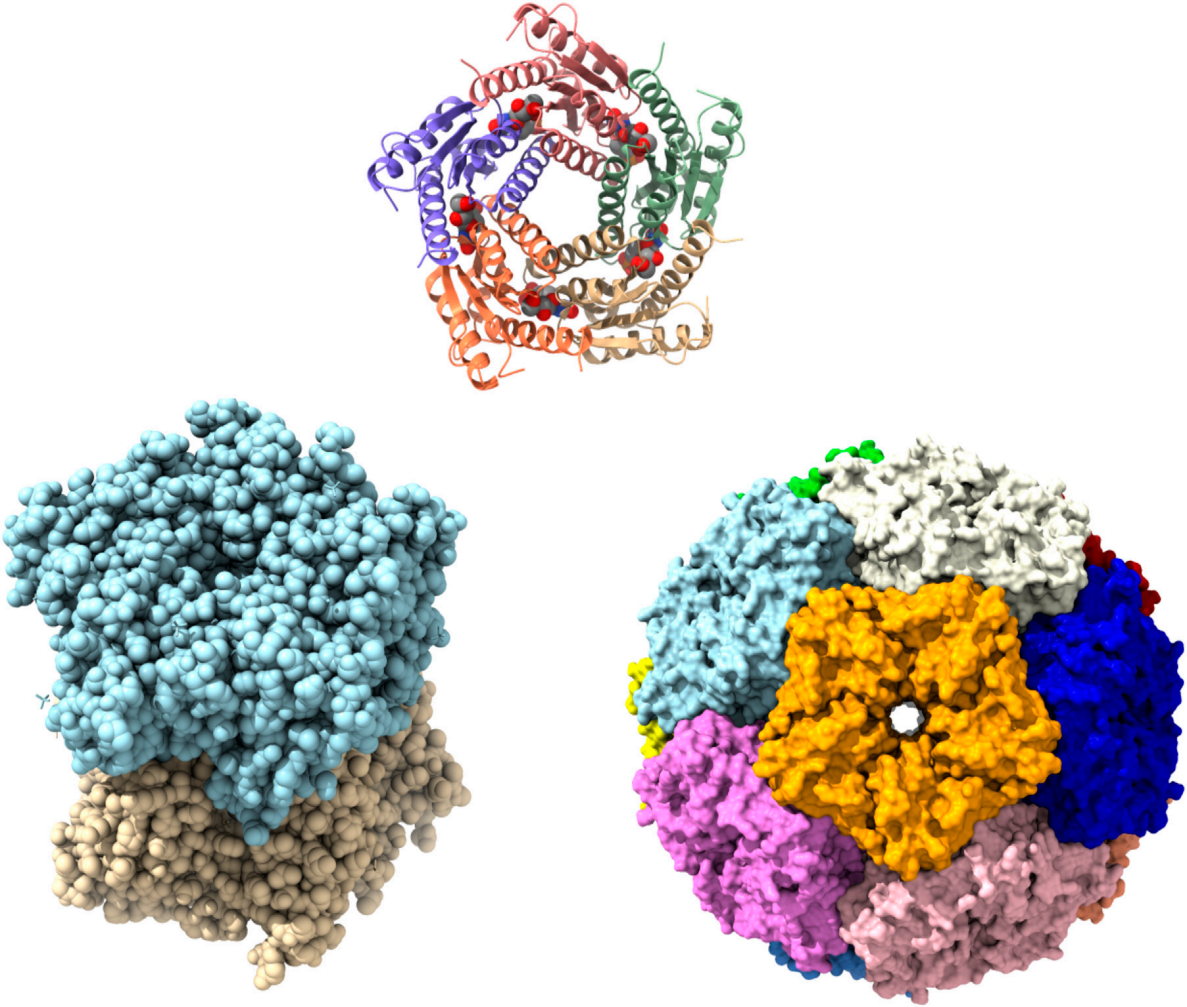
Structures of lumazine synthase (LS) in several oligomeric states. Pentamer assembly of LS from *M. tuberculosis* bound to inhibitor TS50 (5-(1,3,7-trihydro-9-day-ribityl-2,4,8-purinetrione-7-yl)pentane 1-phosphate) at active sites (PDBID: 2C94). Each subunit is represented in different colour (upper). Crystal Structure of LS from *B. abortus* (PDBID: 1XN1) showing as dimer of pentamer (lower left). Surface representation of icosahedral assembly of *S. typhimurium* LS showing each pentamer in different colour (PDBID: 3MK3) (lower right).

The crystal structures of LS from different organisms were reported. All the structural and oligomeric studies performed suggest a pentameric assembly as a basic unit, although, various degrees of the quaternary higher oligomeric states were observed for different organisms. The LS proteins were reported in the pentameric form in *Saccharomyces cerevisiae*, *Schizosaccharomyces pombe*, *M. grisea*, and *M. tuberculosis* (Persson et al., 1999; Meining et al., 2000; Gerhardt et al., 2002a; Morgunova et al., 2005), as dimers of pentamers in *Brucella abortus* (Braden et al., 2000; Zylberman et al., 2004) and as icosahedral capsids consisting of 60 identical subunits, which can be designated as dodecamers of pentamers in *B. subtilis*, *Aquifex aeolicus*, *S. oleracea* and *S. typhimurium* (Schott et al., 1990; Ladenstein et al., 1994; Persson et al., 1999; Zhang et al., 2001; Kumar et al., 2011). Moreover, in *B. subtilis* a new arrangement of LS and RS was seen, they exist as a 1 MDa protein complex having LS forming the outer capsid, while 3 RS subunits occupied the core (Ladenstein et al., 1988; Schott et al., 1990). The mega-dalton protein complex of LS and RS proposed to improve the catalytic productivity of the two processes carried by these two enzymes by substrate channeling especially at low substrate concentrations (Ritsert et al., 1995). The topology of the higher oligomeric form (icosahedral and decameric forms of protein) looks like that of the homopentameric LSs (Figure 5). Comparative analysis of several crystal structures of LS from various species displayed a flavodoxin-like fold irrespective of the oligomeric status, either pentameric or higher quaternary structures of the protein. The overall architecture of the single subunit contains a four-stranded beta-sheet with alpha helices covering the central beta strands. Structural studies of LS structures reveal the topologically equivalent active sites and were located between adjacent subunits creating a cavity to accommodate the substrate (Figure 5). LS binds two substrates, namely, DHBP and ArPP and their location were identified through several structural studies of the protein-ligand complexes formed between the protein and metabolically stable substrate, intermediate, and product analogues (Ritsert et al., 1995; Persson et al., 1999; Meining et al., 2000; Morgunova et al., 2005).

### Riboflavin synthase (RS)

RS is an ultimate protein in the biosynthetic pathway of riboflavin, that catalyzes the dismutation of two DRL to yield one riboflavin and one molecule of ArPP (PLAUT, 1963; Harvey and Plaut, 1966). In essence, it performs the transfer of a butane unit (4-carbon unit) from one molecule of DRL to another DRL molecule and in the process creating riboflavin and ArPP, hence outlining the terminal reaction of the riboflavin biosynthetic pathway. The second product of the reaction, ArPP, can be recycled and serve as a substrate for LS, the penultimate enzyme in the biosynthetic pathway of riboflavin. The enzyme-catalyzed and the uncatalyzed reactions proceed with identical regiochemistry involving a head-to-tail arrangement of the two 4-carbon moieties from which the xylene ring of riboflavin is formed (Paterson and Wood, 1969; Sedlmaier et al., 1987; Ladenstein et al., 2013). The enzyme-catalyzed reaction necessitates the simultaneous occurrence of two substrates in an anti-parallel orientation at the active site of the enzyme. A proposed reaction mechanism of RS proceeds through a pentacyclic intermediate structure, which is formed by the dimerization of DRL and the sequence of two elimination reaction that converts the pentacyclic intermediate into equimolar amounts of riboflavin and ArPP (Illarionov et al., 2001; 2005).

**FIGURE 6:**
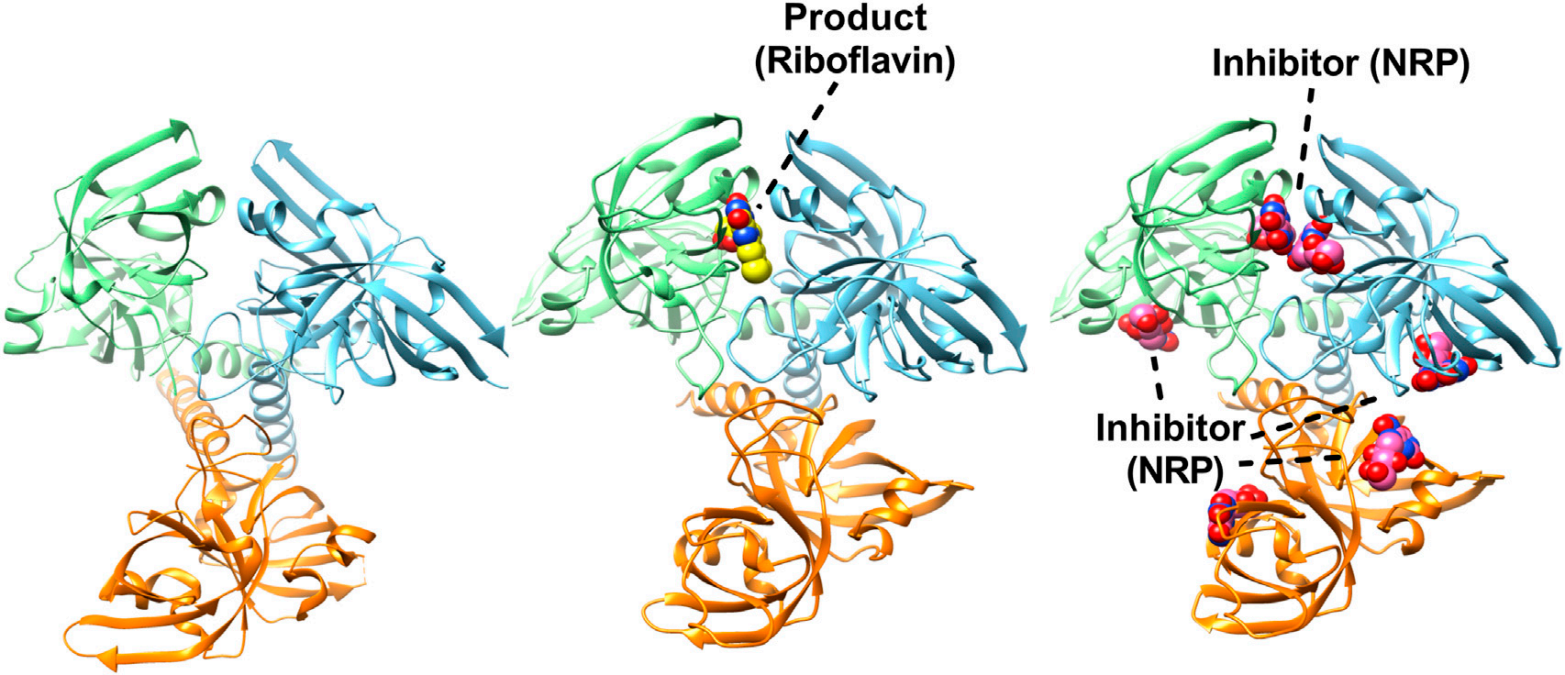
Structures of trimeric riboflavin synthase (RS). Crystal structure of RS from *Escherichia coli* (PDBID: 1I8D). Each subunit is shown in different colour (left). Structure of trimeric RS from *B. abortus* in complex with one riboflavin (PDBID: 4E0F) (middle). Crystal structure of RS from *B. abortus* in complex with 5-Nitro-6-(D-Ribitylamino)-2.4(1H, 3H) Pyrimidinedione (PDBID: 4GQN) highlighting the inhibitor binding in active sites (right).

RS from bacteria, yeast, and plants are shown to form homotrimers (Bacher et al., 1980; Liao et al., 2001b; Gerhardt et al., 2002b), while, in archaea, it forms homopentamer with completely different protein sequences (Ramsperger et al., 2006). In all the RS studied so far, the homotrimeric RS subunits contain intramolecular sequence similarity possibly due to two molecules of similar substrate. The comparison of proximal and distal domains of homotrimeric RS showed a high structural similarity, suggesting that both domains have grown from a common ancestor probably through gene duplication. The three-dimensional structure of RS from *E. coli*, *B. abortus*, and *S. pombe* was determined and showed that the protein was a homotrimer. Each subunit consists of two repeating β-barrel domains that share high sequence and structural similarity. The only difference between the two β-barrels is the presence of a C-terminal helix at the C-terminal β-barrel which is suggested to play a role in the trimerization of RS (Liao et al., 2001b; Gerhardt et al., 2002b; Serer et al., 2014). The structural studies on RS also revealed that the active site is present between the N-terminal β-barrel of one subunit and the C-terminal β-barrel of another subunit with its substrate binding in anti-parallel orientation. The interactions between the subunits of the trimer are primarily mediated by the arrangement of the C-termini (which do not participate in the intra-subunit sequence similarity) in a triple helical motif (Figure 6). The trimeric RS from *E. coli* was solved in the apo form, while RS from *S. pombe* was solved complex with substrate analogue (Liao et al., 2001b; Gerhardt et al., 2002b). The homotrimeric structure of RS from *B. abortus* was solved in the presence of riboflavin and in complex with two product analogues, namely, roseoflavin and 5-nitro-6-ribitylamino-2.4(1H, 3H)-pyrimidinedione (Serer et al., 2014) (Figure 6). Additionally, the crystal structure of N-terminal domain of *E. coli* RS in the presence of riboflavin was solved and showed that it can bind riboflavin and exists as a homodimer which could be superposed with a single subunit of full-length enzyme. This model was used to find out the differences in substrate and product binding at the C and N-terminal domains and validated the proposed reaction mechanism (Meining et al., 2003).

### Inhibitors of riboflavin pathway enzymes

The compound 4-phosphoerythronohydroxamic acid (4PEH) was studied for its inhibitory ability due to structurally mimicking the substrate Ru5P of DHBPS. The compound was found to inhibit DHBPS activity with a Ki value of around 100 μM (Islam et al., 2015). Apart from DHBPS, 4PEH was shown to act as a competitive inhibitor for *M. tuberculosis* ribose-5-phosphate isomerase B (Roos et al., 2005). Structure determination with inhibitor confirms the competitive mechanism where it occupies the active site and was unable to accommodate an intermediate that was critical for DHBPS activity (Islam et al., 2015).

GCH II, being the rate-limiting step in the biosynthesis of riboflavin, makes it a potential novel selective antimicrobial drug target. Phosphomethylphosphonic acid guanyl ester, GMPCPP was known to inhibit the GCH II and act as a GTP substrate analogue. The bound inhibitor structure of *E. coli* GCH II with GMPCPP revealed the detailed stereochemistry of the enzyme active site and helped in the structure-based design of inhibitors of GCH II (Ren et al., 2005). *E. coli* bifunctional deaminase/reductase structure complexed with the substrate analogue ribose-5-phosphate (RP5) was helpful in defining the binding pocket useful for inhibitor design (Stenmark et al., 2007). To gain structural insights and inhibitor design, the structure of *B. subtilis* bifunctional deaminase/reductase in complex with 5-diamino-6-ribosylamino-2.4(1H, 3H)-pyrimidinedione 5′-phosphate (AROPP) was solved at 2.56-Å resolution (Chen et al., 2009). GMP molecule, a substrate analogue for the deaminase reaction was observed in the NADPH-binding site of the reductase domain, occupying the adenine-binding pocket (Dawson et al., 2013). These initial molecules may be exploited to provide starting points for a structure-based approach to antibacterial drug discovery.

Most of the inhibitors were designed or studied centered around LS and RS due to their involvement in the last step of the pathway. The development of LS and RS inhibitors was focused on the modifications of the central pyrimidinedione core, side chain extension from the core and various stable substrates, intermediate and product analogues. Based on the crystal structures complexed with substrate and product of the reaction catalyzed by LS, several organic compounds have been synthesized and characterized by various docking, simulation and spectrophotometrically as inhibitors of LS from *M. tuberculosis*, *B. abortus* and *C. albicans* (Cushman et al., 1998; Braden et al., 2000; Cushman et al., 2001; Cushman et al., 2002a; Cushman et al., 2004; Chen et al., 2005; Cushman et al., 2005; Zhang et al., 2008). The crystal structure of icosahedral *B. subtilis* LS capsids with 5-nitro-6-ribitylamino-2.4-(1H, 3H) pyrimidinedione, a substrate analogue inhibitor, was solved allowing a detailed description of the substrate analogue binding site (Ritsert et al., 1995). Based on the structure-based drug designing, several substrate analogous inhibitors of LS from *C. albicans* have been synthesized, namely, 1,3,7-Trihydro-9-day-ribityl-2,4,8-purinetrione-7-yl (TS13); 3-(1,3-dihydro-9-day-ribityl-2,4,8-purinetrione-7-yl)propane 1-phosphate (TS44); 4-(6.7(5H, 8H)-dioxo-8-day-ribityllumazine-5-yl)butane 1-phosphate (GJ43); and [4-(6-chloro-2,4-dioxo-1,2,3,4 tetrahydropyrimidin-5-yl)butyl] phosphate (JC33). The binding affinities of these inhibitors were measured using isothermal titration calorimetry and were found to be in the micromolar range (Morgunova et al., 2007). Several amide derivatives of 5-phosphonoalkyl-6-D-ribitylaminopyrimidinedione were synthesized and their selectivity for inhibition for LS as well as RS was evaluated. These amide derivatives showed better inhibitory potency to RS than LS and this was further validated by molecular docking demonstrating that the active site of the RS protein could gladly harbor two substrate/substrate analogue/inhibitor molecules (Cushman et al., 2002b).

Several phosphonate inhibitors of LS were manufactured in that the phosphorus atom was separated from the pyrimidinedione ring by a carbon linker. These inhibitors were metabolically stable analogues of intermediate structure designed using enzyme-inhibitor complex. These phosphonates show remarkable LS inhibitory potency with Ki in the range of 100–500 μM (Cushman et al., 1999c; 1999b). Three bis(6,7-dimethyl-8-D-ribityllumazines) containing two lumazine moieties and (ribitylamino) uracils bearing fluorosulfonyl, sulfonic acid, and carboxylic acid were synthesized. These compounds showed very weak inhibitors of RS, although more potent for LS (Cushman et al., 1997; 1999a).

The inhibitors created by the replacement of the pyrimidinedione core with a purine showed inhibition for *B. subtilis* LS and *E. coli* RS in mid micromolar range which further improved to lower micromolar range upon the addition of a ketone group (Cushman et al., 1998; Cushman et al., 2001). A series of inhibitors based on 8-aza derivative with alkyl and phosphate moiety were synthesized. All the synthesized compounds were exceptionally potent inhibitors of LS of *M. tuberculosis*, *M. grisea*, *C. albicans*, and *S. pombe* with inhibition potency in the low nanomolar to sub nanomolar range (Zhang et al., 2007). The designs of dual inhibitors were rapidly progressed because of their effectiveness against LS and RS and the fact it would be rare to mutate both enzymes simultaneously to create a resistant pathogen. Several dual inhibitors were synthesized and found to be effective in inhibiting the LS and RS of various organisms (Cushman et al., 2002a; Cushman et al., 2005; Talukdar et al., 2012).

A robust high-throughput screening (HTS) platform was developed for screening the inhibitors against DHBPS, LS, and RS (Kaiser et al., 2007; Talukdar et al., 2009; Zhao et al., 2009). Covalent hydrates of trifluoromethylated Pyrazoles were recognized as inhibitors of RS, and showed potent antimicrobial activity against *M. tuberculosis* (Zhao et al., 2009). Recently, HTS approach has identified ten molecules from initial 44,000 low molecular weight compounds against RS from *Brucella* spp. With inhibition in the low micromolar range. Several of the most effective inhibitors were subsequently optimized and represent a promising and effective antimicrobial for brucellosis (Serer et al., 2019). Table 1 summarizes all the inhibitors of riboflavin biosynthetic enzymes mentioned in this section.

**TABLE 1:** List of inhibitors (substrate, intermediate and product analogues) for riboflavin biosynthetic enzymes.

Enzymes	Inhibitors	References
3,4-dihydroxy-2-butanone 4-phosphate synthase (DHBPS)	4-phosphoerythronohydroxamic acid (4PEH)	Islam et al. (2015)
GTP cyclohydrolase II (GCH II)	Phosphomethylphosphonic acid guanylyl ester, GMPCPP	Ren et al. (2005)
Pyrimidine deaminase/reductase	Ribose-5-phosphate (RP5)	Stenmark et al. (2007)
	5-diamino-6-ribosylamino-2.4(1H,3H)-pyrimidinedione 5′-phosphate (AROPP)	Chen et al. (2009)
	Guanosine monophosphate (GMP)	Dawson et al. (2013)
Lumazine synthase (LS)	1.5,6,7-Tetrahydro-6,7-dioxo-9-day-ribitylaminolumazines bearing alkyl phosphate Substituents (Ribitylamino)uracils bearing fluorosulfonyl, sulfonic Acid, and carboxylic Acid	Cushman et al. (2005)
	2,6-dioxo-(1H,3H)-9-N-ribitylpurine	Cushman et al. (1997)
	2,6-dioxo-(1H,3H)-8-aza-9-N-ribitylpurine	Cushman et al. (1998)
	6-(6-D-ribitylamino-2,4-dihydroxypyrimidine-5-yl)-1-hexylphosphonic acid	Cushman et al. (1998)
	9-day-Ribitylamino-1.3,7,9-tetrahydro-2,6,8-purinetriones bearing alkyl phosphate and α,α-difluorophosphonate Substituents	Cushman et al. (1999b)
	6-Carboxyalkyl and 6-phosphonoxyalkyl derivatives of 7-Oxo-8-ribitylaminolumazines	Cushman et al. (2004)
	1,4-bis [1-(9-D-ribityl-1,3,7-trihydropurine-2,6,8-trionyl)]butane	Cushman et al. (2002a)
	5-nitro-6-ribitylamino-2.4-(1H,3H) pyrimidinedione	Cushman et al. (2001)
	1,3,7-Trihydro-9-day-ribityl-2,4,8-purinetrione-7-yl (TS13)	Ritsert et al. (1995)
	3-(1,3-dihydro-9-day-ribityl-2,4,8-purinetrione-7-yl)propane 1-phosphate (TS44)	Morgunova et al. (2007)
	4-(6.7(5H,8H)-dioxo-8-day-ribityllumazine-5-yl)butane 1-phosphate (GJ43) [4-(6-chloro-2,4-dioxo-1,2,3,4 tetrahydropyrimidin-5-yl)butyl] phosphate (JC33)	Morgunova et al. (2007)
	3-Alkyl phosphate derivatives of 4.5,6,7-tetrahydro-1-day-ribityl-1H-pyrazolo [3,4-day]pyrimidinedione	Morgunova et al. (2007)
	N-[2,4-dioxo-6-day-ribitylamino-1.2,3,4-tetrahydropyrimidin-5-yl]oxalamic acid derivatives	Morgunova et al., 2007 Zhang et al., 2007 Zhang et al., 2008
Riboflavin synthase (RS)	1.5,6,7-Tetrahydro-6,7-dioxo-9-day-ribitylaminolumazines bearing alkyl phosphate Substituents	Cushman et al. (2005)
	Bis(6,7-dimethyl-8-D-ribityllumazines)	Cushman et al. (1999a)
	2,6-dioxo-(1H,3H)-9-N-ribitylpurine	Cushman et al. (1998)
	2,6-dioxo-(1H,3H)-8-aza-9-N-ribitylpurine	Cushman et al. (1998)
	9-day-Ribitylamino-1.3,7,9-tetrahydro-2,6,8-purinetriones bearing alkyl phosphate and α,α-difluorophosphonate Substituents	Cushman et al. (2004)
	5-phosphonoalkyl-6-D-ribitylaminopyrimidinediones (Amide derivatives)	Cushman et al. (2002b)
	N-[2,4-dioxo-6-day-ribitylamino-1.2,3,4-tetrahydropyrimidin-5-yl]oxalamic acid derivatives	Zhang et al. (2008)

## Conclusion

Structures of all the enzymes of the riboflavin biosynthetic pathway were solved from several bacterial pathogens. High-resolution structures along with enzyme kinetics were helpful in finalizing the reaction mechanism with the formation of various possible intermediates. Structure-based drug designing has started due to the abundant complex structures of these enzymes with substrate, substrate analogs, intermediates, product and product analogs. Several stable compounds were synthesized based on modification of the pyrimidine core, phosphate, and alkyl chain especially for LS and RS. The inhibitors were tested on various microorganisms and found to be effective which provides a template to generate new lead compounds in the development of therapeutics.
