# Supplementary material for: Inhibitors of riboflavin biosynthetic pathway enzymes as potential antibacterial drugs
Source: Front Mol Biosci. 2023 Jul 11;10:1228763. doi: 10.3389/fmolb.2023.1228763 (PMC10366380; doi:10.3389/fmolb.2023.1228763)
Supplement: Supplementary file 1 [file Table1.DOCX]

Table 1: List of inhibitors (substrate, intermediate and product analogues) for riboflavin biosynthetic enzymes.

| **Enzymes** | **Inhibitors** | **References** |
| --- | --- | --- |
|  |  |  |
| 3,4-dihydroxy-2-butanone 4-phosphate synthase (DHBPS) | 4-phosphoerythronohydroxamic acid (4PEH) | (Islam et al., 2015) |
| GTP cyclohydrolase II (GCH II) | Phosphomethylphosphonic acid guanyl ester, GMPCPP | (Ren et al., 2005) |
| Pyrimidine deaminase/reductase | Ribose-5-phosphate (RP5)  5-diamino-6-ribosylamino-2,4(1H,3H)-pyrimidinedione 5′-phosphate (AROPP)  Guanosine monophosphate (GMP) | (Stenmark et al., 2007)  (Chen et al., 2009)  (Dawson et al., 2013) |
| Lumazine synthase (LS) | 1,5,6,7-Tetrahydro-6,7-dioxo-9-d-ribitylaminolumazines bearing alkyl phosphate Substituents  (Ribitylamino)uracils bearing fluorosulfonyl, sulfonic Acid, and carboxylic Acid  2,6-dioxo-(1H,3H)-9-N-ribitylpurine  2,6-dioxo-(1H,3H)-8-aza-9-N-ribitylpurine  6-(6-D-ribitylamino-2,4-dihydroxypyrimidine-5-yl)-1-hexylphosphonic acid  9-d-Ribitylamino-1,3,7,9-tetrahydro-2,6,8-purinetriones bearing alkyl phosphate and α,α-difluorophosphonate Substituents  6-Carboxyalkyl and 6-phosphonoxyalkyl derivatives of 7-Oxo-8-ribitylaminolumazines  1,4-bis[1-(9-D-ribityl-1,3,7-trihydropurine-2,6,8-trionyl)]butane  5‐nitro‐6‐ribitylamino‐2,4‐(1H,3H) pyrimidinedione  1,3,7-Trihydro-9-d-ribityl-2,4,8-purinetrione-7-yl (TS13)  3-(1,3-dihydro-9-d-ribityl-2,4,8-purinetrione-7-yl)propane 1-phosphate (TS44)  4-(6,7(5H,8H)-dioxo-8-d-ribityllumazine-5-yl)butane 1-phosphate (GJ43)  [4-(6-chloro-2,4-dioxo-1,2,3,4 tetrahydropyrimidin-5-yl)butyl] phosphate (JC33)  3-Alkyl phosphate derivatives of 4,5,6,7-tetrahydro-1-d-ribityl-1H-pyrazolo[3,4-d]pyrimidinedione  N-[2,4-dioxo-6-d-ribitylamino-1,2,3,4-tetrahydropyrimidin-5-yl]oxalamic acid derivatives | (Cushman et al., 2005)  (Cushman et al., 1997)  (Cushman et al., 1998)  (Cushman et al., 1998)  (Cushman et al., 1999b)  (Cushman et al., 2004)  (Cushman et al., 2002a)  (Cushman et al., 2001)  (Ritsert et al., 1995)  (Morgunova et al., 2007)  (Morgunova et al., 2007)  (Morgunova et al., 2007)  (Morgunova et al., 2007)  (Zhang et al., 2007)  (Zhang et al., 2008) |
| Riboflavin synthase (RS) | 1,5,6,7-Tetrahydro-6,7-dioxo-9-d-ribitylaminolumazines bearing alkyl phosphate Substituents  Bis(6,7-dimethyl-8-D-ribityllumazines)  2,6-dioxo-(1H,3H)-9-N-ribitylpurine  2,6-dioxo-(1H,3H)-8-aza-9-N-ribitylpurine  9-d-Ribitylamino-1,3,7,9-tetrahydro-2,6,8-purinetriones bearing alkyl phosphate and α,α-difluorophosphonate Substituents  5-phosphonoalkyl-6-D-ribitylaminopyrimidinediones (Amide derivatives)  N-[2,4-dioxo-6-d-ribitylamino-1,2,3,4-tetrahydropyrimidin-5-yl]oxalamic acid derivatives | (Cushman et al., 2005)  (Cushman et al., 1999a)  (Cushman et al., 1998)  (Cushman et al., 1998)  (Cushman et al., 2004)  (Cushman et al., 2002b)  (Zhang et al., 2008) |
